# Editorial: Single-Domain Antibodies—Biology, Engineering and Emerging Applications

**DOI:** 10.3389/fimmu.2018.00041

**Published:** 2018-01-23

**Authors:** Kevin A. Henry, C. Roger MacKenzie

**Affiliations:** ^1^Human Health Therapeutics Research Centre, National Research Council Canada, Ottawa, ON, Canada; ^2^School of Environmental Sciences, University of Guelph, Guelph, ON, Canada

**Keywords:** single-domain antibody, antibody discovery, antibody engineering, therapeutic antibody, diagnostic antibody

**Editorial on the Research Topic**

**Single-Domain Antibodies—Biology, Engineering and Emerging Applications**

Since the discovery of camelid heavy chain-only antibodies (HCAbs) in 1993 (1) and shark immunoglobulin new antigen receptors (IgNARs) in 1995 (2), and the subsequent recognition that the variable domains of these antibodies (V_H_Hs and V_NAR_s, respectively) function autonomously as single-domain antibodies (sdAbs), sdAbs have found many uses across diverse fields. Early work on naturally-occurring antibodies bearing single variable domains spurred renewed interest in the development of human sdAbs, namely, the light chain variable domains (V_L_s) and the heavy chain variable domains (V_H_s) of human conventional antibodies; the first reported sdAbs were actually V_H_s (3). Synthetic human sdAbs are expected to be less immunogenic than V_H_Hs or V_NAR_s but their development is more challenging, requiring steps to ensure the selection of molecules with good biophysical properties and appropriate affinity. There are several advantages offered by sdAbs over conventional antibodies in a wide variety of diagnostic, research, and therapeutic applications, most notably their ease of production in microbial systems, their potential ability to target cryptic epitopes that are inaccessible to larger molecules, and the fact that they can be readily formatted into more complex molecules. This collection brings together 26 reviews and original research articles that together provide extensive coverage of the developments, opportunities, and challenges associated with this unique class of molecules.

## Biology of Single-Domain Antibodies

Although no manuscripts describe the immunobiology of sdAbs themselves, Arbabi-Ghahroudi provides a historical perspective on the discovery and development of camelid sdAbs and highlights how much of the molecular ontogeny of sdAbs still remains poorly understood. Nonetheless, sdAbs have proven to be valuable research tools, especially for studying cellular biology, and two reviews summarize applications of sdAbs in fundamental research. Beghein and Gettemans review and thoroughly assess the current status of sdAbs as research tools in three main areas: (i) the preparation and use of labeled sdAbs in fluorescent microscopy, (ii) the application of sdAbs to the study of protein–protein interactions, and (iii) the use of sAbs as an alternative to RNAi in exploring protein function. They also touch on the usefulness of sdAbs as protein crystallization chaperones in structural biology. Traenkle and Rothbauer briefly review recent development of sdAbs for advanced cellular imaging (“chromobodies”) with a focus on (i) live-cell imaging to visualize the dynamics of cytoskeletal proteins and nuclear components and (ii) the advantages and challenges of using sdAbs in super-resolution microscopy. The ability of sdAbs to be expressed in mammalian cell cytosol and to tolerate fusion with a variety of tags are pivotal in these applications.

## Discovery, Engineering, and Characterization of Single-Domain Antibodies

One methods article, one original research article and one technology report deal with new approaches to camelid sdAb discovery. Historically, most sdAbs have been isolated by selection from *in vitro* display libraries, yielding binders with desired properties to a target antigen. Deschaght et al. applied a next-generation DNA sequencing (NGS) approach to the identification of antigen-specific sdAbs from a phage-displayed V_H_H library constructed from the lymphocytes of an immunized llama. NGS analysis of sdAb-displaying phage enriched after a single round of panning against RON receptor tyrosine kinase correctly identified 35 known binders as well as a large diversity of functional sdAbs that were missed by conventional screening of the same library. Hussack et al. describe the application of an anti-bovine serum albumin (BSA) V_H_H with unique properties (“ABTAG”) to the medium-throughput affinity screening of sdAbs by surface plasmon resonance. The authors found that sdAb–ABTAG dimers bound to a BSA surface could be completely dissociated using low pH, over multiple cycles, without loss of surface activity, and exploited this to recover rare ultra-high-affinity V_H_Hs against CEACAM6 that were missed by panning of a phage-displayed V_H_H library. Eden et al. describe protocols they have developed for DNA immunization of camelids and identification of sdAbs against membrane proteins; historically, this has been an arduous and unreliable process in large outbred animal species.

One original research article and one technology report describe new approaches to the isolation of human sdAbs. Henry et al. designed a set of novel phage-displayed sdAb libraries, constructed by synthetic randomization of rare fully human autonomous V_H_ and V_L_ domains, and provide a molecular explanation for the variable success rates in obtaining antigen-specific binders from such libraries: in the absence of solubilizing framework mutations akin to those of camelid V_H_Hs, fully human sdAbs rely heavily on their CDR sequences both for stability and solubility as well as for binding, imposing fundamental limitations on the sequences of these molecules. One potential workaround is to increase throughput, and Drabek et al. describe an automatable high-throughput technology for isolating fully human, soluble, high-affinity antigen-specific HCAbs. Building upon a previous generation of transgenic mice bearing hybrid llama-human *igh* loci (llama V_H_H genes; human D, J, and C genes), the authors have constructed a new transgenic mouse line (4HVH) whose *igh* locus contains germline human V_H_, D, and J genes as well as human C genes lacking the C_H_1 exon. HEK293T cells in microtiter plates are transfected with DNA encoding HCAbs derived from bone marrow and spleen plasma cells of immunized mice and positive clones identified by ELISA. The method thus relies on the apparatus of the murine immune system to rearrange, select, and affinity mature human V_H_ domains with good biophysical properties.

One original research paper and one review deal with engineering sdAbs for higher affinity and improved stability, respectively. Tiller et al. describe a novel approach to sdAb affinity maturation involving (i) identification of CDR residues amenable to randomization by computational and experimental alanine scanning mutagenesis, (ii) conservative randomization of permissive positions with a mixture of the wild-type residue and frequent, naturally occurring residues, and (iii) screening of the resulting libraries by yeast display to identify sdAbs bearing combinations of mutations conferring ≥5-fold affinity gains. Careful analysis of each variant revealed that CDR sequence deviation involves complex tradeoffs between sdAb affinity, specificity, and stability. Goldman et al. give a good overview of the literature on engineering sdAbs for improved stability, including (i) strategies to increase the stability of camelid and shark sdAbs (e.g., through framework mutation or introduction of non-canonical disulfide bonds) and (ii) analytical techniques for assessing sdAb stability.

## Single-Domain Antibodies as Diagnostics

Because of their generally high stability and low cost of production in microbial systems, sdAbs have been viewed as potentially superior alternatives to conventional antibodies in diagnostic applications. One review and two original research articles focus on diagnostic applications of sdAbs. Gonzalez-Sapienza et al. summarize the pros and cons of sdAbs in analytical and diagnostic applications and review recent developments that highlight the potential of sdAbs in immunodetection technologies. Stijlemans et al. review the use of sdAbs as reagents for the diagnosis and treatment of African trypanosomiasis (AT). Current diagnostic procedures for AT are inadequate, and there is no effective vaccine; chemotherapy is the only treatment but involves high drug toxicity and increasing drug resistance. Anti-AT sdAbs have shown promise for the detection of several parasite antigens as well as for targeted drug delivery, and may even exert Fc-independent trypanolytic activity. Harmsen et al. report on their latest efforts to isolate V_H_Hs for use in quality control of foot-and-mouth disease vaccines. Only intact inactivated viral particles are efficacious vaccine antigens but intact virus can dissociate to yield ineffective capsid degradation products; reagents specific for intact virus and broadly cross-reactive with multiple viral strains are therefore needed.

## Single-Domain Antibodies as Therapeutics

Two reviews and one perspective article on various aspects of tumor imaging and tumor targeting highlight the intense interest in the development of sdAbs for cancer therapy. Xenaki et al. provide a cell biologist’s perspective on the factors (primarily relating to molecular size and binding properties) that govern intratumoral uptake and distribution of antibodies and antibody fragments. Hu et al. review recent progress in developing sdAbs as targeting modules for drug delivery systems, including (i) toxins, enzymes, and cytokines; (ii) liposomes, extracellular vesicles, micelles, microbubbles, and nanoparticles; and (iii) viral vectors. They also discuss emerging technologies for intracellular delivery of sdAbs and sdAbs as tools for molecular imaging. Arezumand et al. review the development of anti-angiogenic sdAbs for diagnosis and treatment of cancer.

In the development of therapeutic antibodies, an advantage of sdAbs is the relative ease of reformatting them into more complex and efficacious molecules. Nosenko et al. offer a brief perspective on their efforts to develop bispecific TNF inhibitors using sdAbs: one arm of these molecules binds and neutralizes TNF-α, while the second arm targets the effects of the antibody to specific populations of macrophages and monocytes. Two original research articles from Ablynx illustrate the potential of linking two or more sdAbs using polypeptide linkers. Beirnaert et al. elucidated the crystal structures of three V_H_Hs in complex with TNF. The structures revealed the molecular basis of the very high neutralization potency of a heterodimeric construct in which two of the V_H_Hs recognizing distinct epitopes are linked by a nine residue linker: the biparatopic V_H_H:V_H_H heterodimer engages both arms in an intramolecular fashion on a single TNF molecule and blocks two of three receptor-binding sites, but only in one of the two possible V_H_H orientations. Similarly, Desmyter et al. developed high-affinity V_H_Hs against IL23, and, based on the crystal structures of three V_H_Hs in complex with IL23, rationally designed multivalent sdAb dimers with high IL23 neutralization activity using molecular linkers of appropriate length. A biparatopic sdAb-based heterotrimer in which two anti-IL23 V_H_Hs flanked an anti-human serum albumin (HSA) V_H_H (for serum half-life extension) was a more potent neutralizer than any single anti-IL23 V_H_H fused to the same albumin binder.

Three original research articles from Elasmogen demonstrate the modularity and therapeutic utility of shark V_NAR_s. Kovaleva et al. report that anti-ICOSL V_NAR_s selected from an immune nurse shark phage display library and reformatted as Fc-fusions markedly reduced inflammation in a mouse model of inflammatory eye disease, uveitis, when administered systemically. The observation that V_NAR_s, but not IgGs and V_NAR_–Fcs, could penetrate the cornea when applied topically at high concentration raises the hope that these V_NAR_s (in multivalent formats lacking Fc) might be useful for topical treatment of uveitis. Extending the results of Beirnaert et al. using llama V_H_Hs, Ubah et al. isolated immune shark V_NAR_s against TNF and reformatted them, first as V_NAR_:V_NAR_ homo- and heterodimers and then as tetravalent biparatopic V_NAR_–Fcs. Even in the absence of structural information, the authors were able to achieve a ~50,000-fold improvement in the neutralizing potency of the V_NAR_ monomer through molecular reformatting. Steven et al. optimized two previously humanized versions of an anti-HSA V_NAR_ domain which had less desirable biophysical properties than the parental V_NAR_. After random mutagenesis of the two humanized domains and a phage display selection process, mutants with acceptable biophysical properties were obtained without compromising affinity or species cross-reactivity and extended serum half-life in a rat PK model. When fused in tandem with other humanized V_NAR_ moieties, the optimized anti-HSA V_NAR_ (BA11) should be useful for clinical studies.

Finally, Tian et al. from Helix BioPharma describe the optimized construction of an immunoconjugate in which an anti-VEGFR2 V_H_H is linked to urease. The sdAb targets the immunoconjugate to tumor vasculature, where urease enzymatically converts endogenous urea to ammonia resulting in both direct and indirect antitumor effects. A similar conjugate targeting a non-small-cell lung cancer antigen is currently in clinical trials (http://www.helixbiopharma.com/).

## Antiviral Single-Domain Antibodies

One review and two original research articles describe applications of sdAbs directed against viruses in basic research, diagnostics, and biodefense. Wu et al. review the current state of knowledge on sdAb targeting and neutralization of human-tropic viruses and illustrate the potential of sdAbs to probe different sets of epitopes on viral glycoproteins compared to conventional antibodies. Darling et al. investigated the ability of tandem sdAb dimers directed against repetitive epitopes of filoviral nucleoproteins (“Xintrabodies” or cross-linking intrabodies) to cross-link capsid proteins and inhibit viral replication. Minimal amounts of Xintrabody ablated nucleoprotein incorporation into viral particles despite the presence of large amounts of nucleoprotein in the cytoplasm. In a companion manuscript, Garza et al. present the crystal structures of three of the sdAbs in complex with the nucleoprotein of Marburg virus, an agent causing viral hemorrhagic fever and a bioterror threat. The sdAbs bind to a cryptic epitope, a three-helix structure at the nucleoprotein C-terminus that has been conserved over 50 years of virus evolution. Engagement with these sdAbs gave the first crystal structure of the nucleoprotein C-terminus and identified an epitope that should be useful for diagnostic purposes and, possibly, intrabody-based countermeasures.

## Final Thoughts

Single-domain antibodies, once considered immunological oddities of minor academic interest, have become critical tools in fundamental research as well as in the design of biologic drugs. Regulatory approval of the first sdAb-based drug (anti-vWF caplacizumab) is expected shortly, possibly in 2018, and may substantially alter perceptions and attitudes toward these molecules in the medical and scientific communities.

The editors would like to thank all contributors for the many excellent submissions to this Research Topic, as well as the reviewers and the *Frontiers in Immunology* editorial office.

## Author Contributions

KAH and CRM wrote the manuscript and approved it for publication.

## Conflict of Interest Statement

The authors declare that the research was conducted in the absence of any commercial or financial relationships that could be construed as a potential conflict of interest.
